# Thyroid Cancer Incidence and Trends in United States and Canadian Pediatric, Adolescent, and Young Adults

**DOI:** 10.3390/cancers17091429

**Published:** 2025-04-24

**Authors:** May Z. Gao, Tariq M. Omer, Katherine M. Miller, Matthew C. Simpson, Aleksandr R. Bukatko, Kalipa Gedion, Eric Adjei Boakye, Karen M. Kost, James A. Dickinson, Mark A. Varvares, Nosayaba Osazuwa-Peters

**Affiliations:** 1 Duke University School of Medicine, Durham, NC 27710, USA; may.gao@duke.edu (M.Z.G.); tariq.omer@duke.edu (T.M.O.); 2 Department of Pediatric Otolaryngology, Head and Neck Institute, Cleveland Clinic Foundation, Cleveland, OH 44195, USA; polednk@ccf.org; 3 Department of Otolaryngology–Head and Neck Surgery, Saint Louis University School of Medicine, St. Louis, MO 63104, USA; matthew.simpson@health.slu.edu (M.C.S.); arbukatko@gmail.com (A.R.B.); 4 Duke Global Health Institute, Durham, NC 27710, USA; kalipa.gedion@duke.edu; 5 Department of Public Health Sciences, Henry Ford Health System, Detroit, MI 48202, USA; eadjei1@hfhs.org; 6 Department of Otolaryngology–Head and Neck Surgery, Henry Ford Health System, Detroit, MI 48202, USA; 7 Henry Ford Health + Michigan State University Health Sciences, Detroit, MI 48202, USA; 8 Department of Epidemiology and Biostatistics, Michigan State University College of Human Medicine, East Lansing, MI 48824, USA; 9 Department of Otolaryngology–Head and Neck Surgery, McGill University, Montreal, QC H3A 0G4, Canada; karen.kost@mcgill.ca; 10 Departments of Family Medicine and Community Health Sciences, Cumming School of Medicine, University of Calgary, Calgary, AB T2N 4N1, Canada; dickinsj@ucalgary.ca; 11 Department of Otolaryngology, Massachusetts Eye and Ear Infirmary, Harvard Medical School, Boston, MA 02114, USA; mark_varvares@meei.harvard.edu; 12 Department of Head and Neck Surgery & Communication Sciences, Duke University School of Medicine, Durham, NC 27710, USA; 13 Department of Population Health Sciences, Duke University School of Medicine, Durham, NC 27710, USA; 14 Cancer Risk, Detection, and Interception Program, Duke Cancer Institute, Durham, NC 27710, USA

**Keywords:** thyroid cancer, pediatric, adolescent and young adults (AYA), North American Association of Central Cancer Registries’ (NAACCR), incidence trends, cancer surveillance

## Abstract

Background/Objectives: Thyroid cancer incidence has risen in both the United States and Canada, despite differing healthcare systems. While overdiagnosis likely partly explains this trend in adults, its impact on younger populations is unclear. We used the North American Association of Central Cancer Registries, which included 133,808 thyroid cancer cases from the United States and Canada, to assess incidence trends among pediatric, adolescent, and young adult (PAYA) populations. Methods: Age-adjusted incidence rates (AAIR) per 100,000 person-years (PY) were compared using rate ratios (RR), stratified by sex, age, race/ethnicity (United States only), and histology. Joinpoint regression estimated annual percentage changes (APC) and average APCs (AAPC) in AAIRs. From 1995 to 2014, thyroid cancer incidence increased by 137%. Significant increases occurred across all age groups (0–14, 15–24, 25–34, 35–39 years). The rate increase was highest for papillary thyroid cancer (AAPC = 5.50, 95% CI 5.06, 5.94), and among individuals aged 35–39 years (AAPC = 5.99, 95% CI 4.84, 7.15). Of racial/ethnic groups in the United States, non-Hispanic White individuals had the highest AAIR (6.22 per 100,000 PY). Mortality has changed minimally. Conclusions: Over the past two decades, thyroid cancer incidence has increased in individuals under 40. While evidence suggests that overdiagnosis primarily accounts for this trend, other contributing factors cannot be ruled out. Further research and surveillance of the drivers of increased incidence are critical.

## Simple Summary

Thyroid cancer rates have been rising in North America, but most research has focused on adult patients. This study examines trends in thyroid cancer among pediatric, adolescents, and young adults (PAYA) in the United States and Canada. Using a comprehensive North American cancer registry, we found that thyroid cancer incidence increased by 137% from 1995 to 2014, with significant rises across all age groups. The increase was most pronounced in young White females and was highest for papillary thyroid cancer. Our findings suggest that overdiagnosis from thyroid ultrasound and incidental findings from other imaging are occurring, but other factors could be contributing to the rise. These results highlight the need for continued epidemiological monitoring and further research to understand the drivers of thyroid cancer incidence in young populations.

## 1. Introduction

In 2025, there will be about 44,020 new diagnoses of thyroid cancer, making it the ninth most common cancer among women in the United States [1]. In Canada, it is the seventh most common cancer in women, with 6600 new cases in 2024 [2]. Despite the increasing incidence of thyroid cancer, mortality has remained fairly stable globally [3], raising concerns that increased detection—rather than a true rise in disease—may be driving the observed trends. The United States is an exception, where mortality has significantly increased since the 1980s [4,5]. While overdiagnosis has been implicated in rising thyroid cancer rates among adults [6,7], its role in younger populations has been understudied. Some studies posit that factors beyond overdiagnosis—such as environmental exposures, obesity, and genetic predispositions—may also contribute to the increasing incidence, though this evidence remains limited [8,9,10,11]. The interplay between overdiagnosis and these other potential contributors remains unclear, highlighting a critical need for more nuanced analyses that can distinguish between increases in true disease and increases due to surveillance and incidental findings on imaging.

In North America, overdiagnosis is linked mostly to females ≥ 40 years old and papillary histology [9,10]. In contrast, thyroid cancer is relatively rarer among individuals < 40 years old [12]. The American Thyroid Association (ATA) does not recommend asymptomatic thyroid screening until the age of 35 [13], and the Canadian Task Force on Preventive Health Care is against screening asymptomatic nonpregnant adults ≥ 18 years unless there are risk factors for thyroid dysfunction [14]. Screening patterns alone do not explain the increasing incidence in pediatric, adolescent, and young adult (PAYA) populations, for whom there is no recommendation for asymptomatic screening. Diagnostic imaging for other reasons, including ultrasound, CT, and MRI, can lead to the incidental detection of subclinical tumors and subsequently unnecessary treatment and harm [4,15,16].

Moreover, worldwide trends echo these concerns. Several European countries, including France and Italy, have recorded sharp rises in thyroid cancer rates over the past two decades, partially attributed to increased diagnostic scrutiny [11,15]. In Asia, the Republic of Korea has demonstrated one of the most pronounced increases, with women’s thyroid cancer rates increasing nearly fivefold, linked to widespread screening by neck ultrasonography [11,17]. China and Japan have also reported notable upward trends, though at a slower pace [11]. These global observations suggest that enhanced imaging, more aggressive screening, and potentially environmental or lifestyle factors could contribute to the overarching increase in thyroid cancer diagnoses.

Building on previous studies [8,9,18,19], we hypothesized that the incidence of thyroid cancer continues to rise even among PAYA cohorts—groups traditionally considered at lower risk for overdiagnosis. Therefore, we aimed to estimate the incidence trends of thyroid cancer in North America (United States and Canada) among individuals aged 0–39 and determine differences in trends when stratified by sex, age, race, and histological subtype.

## 2. Methods

### 2.1. Study Design

We conducted a population-based, retrospective study using data from the North American Association of Central Cancer Registries (NAACCR) Cancer in North America (CiNA) Public Use dataset [20]. The study period was 1995–2014, and we focused on patients aged 0–39 years with thyroid cancer. This approach allowed us to analyze incidence trends across both the United States and Canada. Institutional review board approval was not required as all data were de-identified and publicly available.

### 2.2. Data Source

The NAACCR CiNA Public Use dataset covers cancer patients in the United States and Canada and is comprised of registries within the National Program of Cancer Registries; Center for Cancer Care & Research Provincial and Territorial Registries; and Surveillance, Epidemiology, and End Results [20]. Only data from Gold and Silver NAACCR-certified registries are included in the CiNA Public Use dataset, ensuring the quality of the data provided.

### 2.3. Study Population

Patients diagnosed with malignant thyroid cancer between 1995 and 2014 at ages 0–39 years were included; malignant thyroid cancer was defined as a primary malignancy of the thyroid (International Classification of Diseases-Oncology, third edition (ICD-O-3) C739) that had any ICD-O-3 histology value, except 9050–9055, 9140, or 9590–9992 [21]. All eligible malignancies were included, including multiple malignancies. To maintain population consistency over the study period, only registries that provided data for all years from 1995 to 2014 were included for analysis. Also, although data from 2015 were available, only data through 2014 were analyzed because 2015 data from Ontario (the most populous Canadian province) were unavailable (Table 1).

### 2.4. Data Analysis

We used SEER*Stat version 8.3.5 (Surveillance Research Program, National Cancer Institute, Bethesda, MD, USA) to calculate overall and yearly age-adjusted incidence rates (AAIRs) for thyroid cancer diagnosed between 1995 and 2014 for Canada, the United States, and both countries combined. For each country individually and combined, AAIRs were stratified by sex (male, female), age based on estrogen exposure [0–14 (pediatrics and pre-pubertal), 15–24 (pre childbearing years), 25–34 (childbearing years based on average age at first child for United States and Canada), 35–39 (potential overdiagnosis group as the ATA recommends screening of thyroid-stimulating hormone (TSH) levels beginning at 35 years) [13,22], and histology/microscopic confirmation (confirmed papillary (ICD-O-3 histology 8050, 8260, 8340–8344, 8350, 8450–8460), confirmed follicular (8290, 8330–8335), confirmed medullary (8345, 8510–8513)/anaplastic (8020–8035)/other, not microscopically confirmed). Also, AAIRs for the United States were stratified by race/ethnicity (Hispanic, non-Hispanic White, non-Hispanic Black, non-Hispanic Asian/Pacific Islander/American Indian/Alaska Native (API/AIAN)). AAIRs by race were not estimated for Canada as race/ethnicity information was not provided. All AAIRs were age adjusted using the 2000 US Standard Population and were reported as incidence per 100,000 person-years (PY). This standard is recommended for population-based cancer incidence studies by the National Cancer Institute and facilitates comparability across different regions and time periods [23]. SEER*Stat computed rate ratios (RR) and 95% confidence intervals (CI) for 1995–2014 overall AAIRs between a reference group and other values for each variable within each country.

Joinpoint Regression Program version 4.6.0.0 (Statistical Methodology and Applications Branch, Surveillance Research Program, National Cancer Institute, Bethesda, MD, USA) estimated time periods with significant increases or decreases in AAIRs using Joinpoint regression models, as well as annual percentage change (APC) and 95% confidence intervals (CI) for rates between Joinpoint years. The final Joinpoint models were based on log-transformed AAIRs to better ensure the normality of residuals [24]. The permutation test method determined the model with the fewest number of Joinpoints necessary to effectively characterize trends [25] with a maximum of 3 Joinpoints. Trends with multiple APCs were summarized with average APCs (AAPC).

For Canada, the United States, and both countries combined, we computed Joinpoint regressions for all thyroid cancer patients combined (including unknown race/ethnicity for United States cases) and by sex, age, histology, and race (excluding unknown race/ethnicity, United States only) groupings. Joinpoint regressions were further computed by sex and age simultaneously, and in the United States, regressions were also computed by race and sex simultaneously. Because CiNA suppresses output for rates based on fewer than six cases, some variable groupings could not have Joinpoint regressions estimated for them. Significance for all tests was set at α = 0.05, and all tests were two-tailed.

We conducted pairwise parallelism tests to assess whether trends in age-adjusted incidence rates significantly differed in slope between subgroups. These comparisons were made across age, sex, histology, and, in the United States, across race/ethnicity and sex-by-race subgroups. We also compared trends between countries (Canada vs. United States) for each relevant subgroup. For example, we compared all unique age group pairs (e.g., 0–14 vs. 15–24), stratified by sex and country when appropriate. Adjusted *p*-values were calculated using the Bonferroni method to account for multiple comparisons. The Supplementary Materials Tables S1 and S2 includes full Joinpoint outputs, AAPC differences with 95% confidence intervals, and adjusted *p*-values for each comparison.

## 3. Results

### 3.1. Demographic Characteristics

This study included 133,808 thyroid cancer tumors for subjects aged between 0 and 39 years in the United States and Canada combined. In Canada, there were 15,492 thyroid cancer cases (11.6%), and in the United States there were 118,316 (88.4%). The sample was 82.4% female, 98.3% 15–39 years (adolescent and young adult), with 90.0% papillary histology. The United States sample was 68.0% non-Hispanic White (Table 1).

### 3.2. Overall Incidence Trend

**Figure 1:**
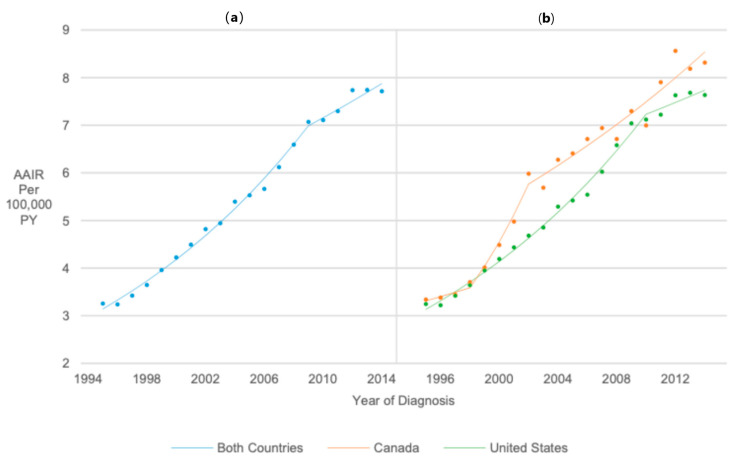
Joinpoint regression trends in age-adjusted incidence of thyroid cancer among pediatrics, adolescents, and young adults in Canada/United States combined (**a**), and from Canada and the United States separately (**b**), from 1995 to 2014.

The overall AAIR of thyroid cancer among the PAYA population in both countries combined (1995–2014) was 5.48 per 100,000 PY (AAIR = 5.48, 95% CI 5.45, 5.51). The 2014 AAIR (7.72 per 100,000 PY) was 137% higher than the 1995 rate (3.26 per 100,000 PY). The AAIR increased 5.87% yearly from 1995 to 2009 (APC = 5.87, 95% CI 5.51, 6.24) (Table 2). From 2009 to 2014, there was a less drastic yearly increase of 2.42% (APC = 2.42, 95% CI 1.11, 3.75). The average annual increase in AAIR from 1995 to 2014 was 4.95% (AAPC = 4.95, 95% CI 4.54, 5.36) (Figure 1).

### 3.3. Temporal Trends in Thyroid Cancer Incidence Rates in the United States vs. Canada

The AAIR in Canada (AAIR = 5.94, 95% CI 5.85, 6.04) was 9.6% higher than in the United States (AAIR = 5.42, 95% CI 5.39, 5.45). In Canada, the 2014 AAIR (8.32 per 100,000 PY) was 149% higher than the 1995 rate (3.34 per 100,000 PY). In the United States, the 2014 AAIR (7.64 per 100,000 PY) was 135% higher than the 1995 rate (3.25 per 100,000 PY). From 1995 to 1998, the AAIR in Canada remained stable, but from 1998 to 2002, the AAIR increased 12.60% annually (APC = 12.60, 95% CI 4.82, 20.96). The AAIR continued to increase at a lower rate from 2002 to 2014 (APC = 3.32, 95% CI 2.63, 4.02). The average of these trends was a 5.11% annual increase (AAPC = 5.11, 95% CI 3.21, 7.05). In the United States, the AAIR increased 5.73% annually from 1995 to 2010 (APC = 5.73, 95% CI 5.41, 6.07) but remained stable from 2010 to 2014. This resulted in an average annual increase of 4.87% (AAPC = 4.87, 95% CI 4.43, 5.32) (Figure 1). The parallelism test comparing AAPCs in Canada and the US indicated that trend changes occurred at different times in the two countries (*p* < 0.01); however, the difference in AAPCs was not significantly different between the countries (AAPC difference = 0.24, 95% CI −1.74, 2.21).

### 3.4. Age at Diagnosis

**Figure 2:**
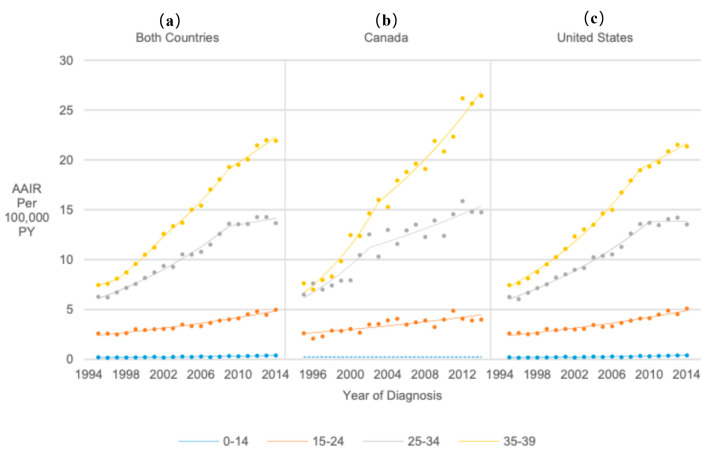
Joinpoint regression trends in age-adjusted incidence of thyroid cancer among pediatrics, adolescents, and young adults in Canada/United States combined (**a**). Canada (**b**), and the United States (**c**) from 1995 to 2014, stratified by age at diagnosis. Groups with dotted lines had at least one year with insufficient data; the dotted line represents their overall age-adjusted incidence rate from 1995 to 2014.

Thyroid cancer incidence was significantly higher among 15–24-year-olds (RR = 13.73, 95% CI 13.15, 14.34), 25–34-year-olds (RR = 40.33, 95% CI 38.69, 42.06), and 35–39-year-olds (RR = 55.66, 95% CI 53.38, 58.06) than in 0–14-year-olds. However, AAIRs for 0–14-year-olds significantly increased by 4.28% annually from 1995 to 2014 (APC = 4.28, 95% CI 3.36, 5.20). AAIRs for 15–24-year-olds significantly increased 3.72% annually from 1995 to 2014 (APC = 3.72, 95% CI 3.34, 4.10). AAIRs for 25–34-year-olds significantly increased from 1995 to 2009 (APC = 5.77, 95% CI 5.30, 6.24) but remained stable from 2009 to 2014, resulting in a significant 4.53% AAPC from 1995 to 2014 (AAPC = 4.53, 95% CI 4.01, 5.06). There were three significant AAIR increases for 35–39-year-olds from 1997 to 2014 after stable rates from 1995 to 1997, resulting in an average annual increase of 5.99% (AAPC = 5.99, 95% CI 4.84, 7.15) (Figure 2).

### 3.5. Sex

**Figure 3:**
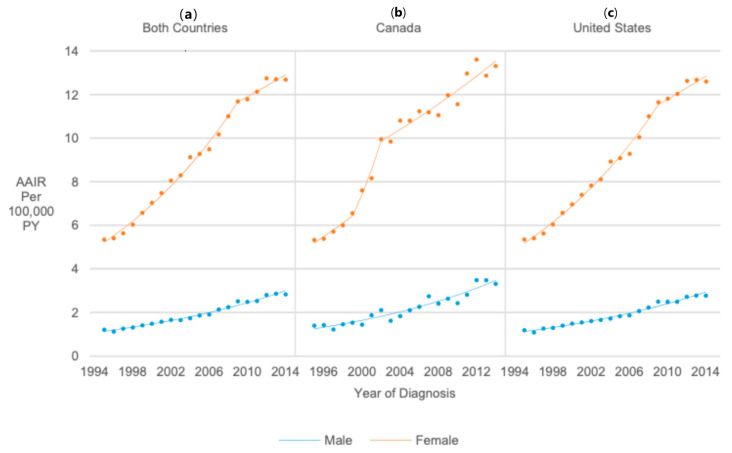
Joinpoint regression trends in age-adjusted incidence of thyroid cancer among pediatrics, adolescents, and young adults in Canada/United States combined (**a**), in Canada (**b**), and in the United States (**c**) from 1995 to 2014 stratified by sex.

Thyroid cancer incidence in both countries combined was over four times higher among females than males (RR = 4.74, 95% CI 4.67, 4.81). AAIRs for males increased 5.22% annually from 1995 to 2014 (APC = 5.22, 95% CI 4.88, 5.55). From 1995 to 2009, AAIRs for females increased 5.92% annually (APC = 5.92, 95% CI 5.53, 6.31), and from 2009 to 2014, AAIRs increased 2.04% annually (APC = 2.04, 95% CI 0.65, 3.45), resulting in an average increase of 4.89% annually (AAPC = 4.89, 95% CI 4.45, 5.32) (Figure 3).

### 3.6. Histology

**Figure 4:**
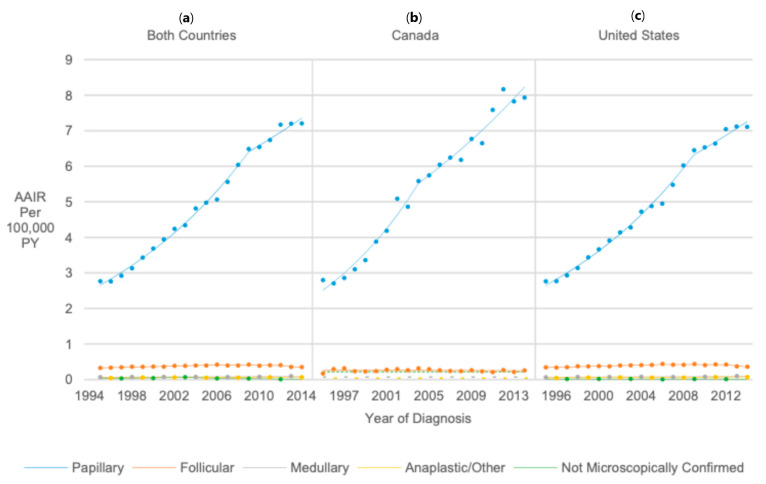
Joinpoint regression trends in age-adjusted incidence of thyroid cancer among pediatrics, adolescents, and young adults in Canada (**b**), the United States (**c**), and both countries (**a**) combined from 1995 to 2014 stratified by histologic type. Groups with dotted lines had at least one year with insufficient data; the dotted line represents their overall age-adjusted incidence rate from 1995 to 2014.

Among the microscopically confirmed histologic types, papillary thyroid cancer had the highest AAIR from 1995 to 2014 (AAIR = 4.93 per 100,000 PY), 13 times higher than follicular (AAIR = 0.38 per 100,000 PY; RR = 0.08, 95% CI 0.07, 0.08), medullary (AAIR = 0.07 per 100,000 PY; RR = 0.02, 95% CI 0.01, 0.02), and anaplastic/other (AAIR = 0.06 per 100,000 PY; RR = 0.01, 95% CI 0.01, 0.01). The increase in rates was highest for papillary (AAPC = 5.50, 95% CI 5.06, 5.94), and less for medullary (AAPC = 1.16, 95% CI 0.47, 1.85), and anaplastic/other histology thyroid cancer (AAPC = 1.94, 95% CI 0.68, 3.22). By contrast, AAIRs for follicular thyroid cancer increased 2% annually from 1995 to 2006 (APC = 2.00, 95% CI 1.52, 2.49), remained stable from 2006 to 2012, and then decreased 7.00% annually from 2012 to 2014 (APC = −7.00, 95% CI −13.33, −0.21), resulting in no significant overall change in AAIRs from 1995 to 2014 (Figure 4).

### 3.7. Race (United States Only)

**Figure 5:**
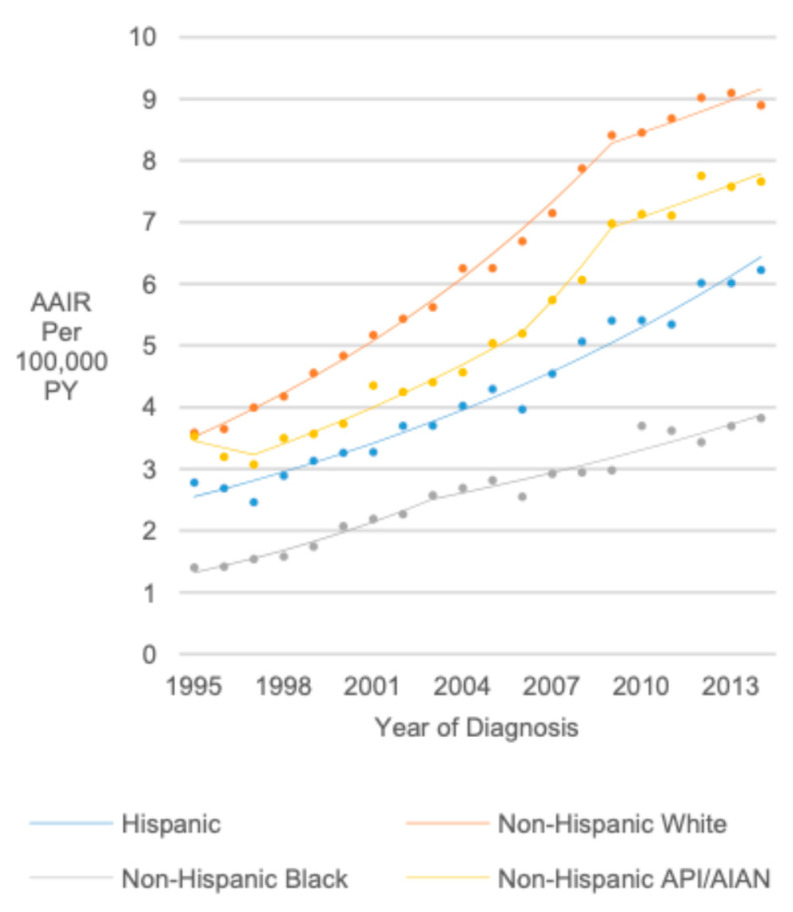
Joinpoint regression trends in age-adjusted incidence of thyroid cancer among pediatrics, adolescents, and young adults in the United States stratified by race/ethnicity.

The highest rates occurred among non-Hispanic White individuals (1995–2014 AAIR = 6.22), followed by non-Hispanic API/AIAN (AAIR = 5.43; RR = 0.87, 95% CI 0.85, 0.89) Hispanic individuals (AAIR = 4.38; RR = 0.70, 95% CI 0.69, 0.71), and non-Hispanic Black individuals (AAIR = 2.59; RR = 0.42, 95% CI 0.41, 0.43). The change in rates was also highest for non-Hispanic White individuals, with a 6.30% annual increase from 1995 to 2009 (APC = 6.30, 95% CI 5.96, 6.64) and a 2% annual increase from 2009 to 2014 (APC = 2.03, 95% CI 0.77, 3.30), resulting in an AAPC of 5.16 (AAPC = 5.16, 95% CI 4.77, 5.55). Non-Hispanic API/AIAN individuals experienced AAIR increases from 1997 to 2006 (APC = 5.47, 95% CI 4.00, 6.96) and 2009 to 2014 (APC = 2.40, 95% CI 0.46, 4.37), with an AAPC of 4.37 (AAPC = 4.37, 95% CI 2.22, 6.56). Among Hispanic individuals, there was a 4.99% annual AAIR increase from 1995 to 2014 (APC/AAPC = 4.99, 95% CI 4.56, 5.43). However, from 1995 to 2003, non-Hispanic Black individuals experienced an 8.36% annual increase (APC = 8.36, 95% CI 5.89, 10.88) and a 4.00% annual increase from 2003 to 2014 (APC = 4.00, 95% CI 2.88, 5.13). This resulted in an AAPC of 5.81% (AAPC = 5.81, 95% CI 4.70, 6.94) (Figure 5).

### 3.8. Sex/Age

**Figure 6:**
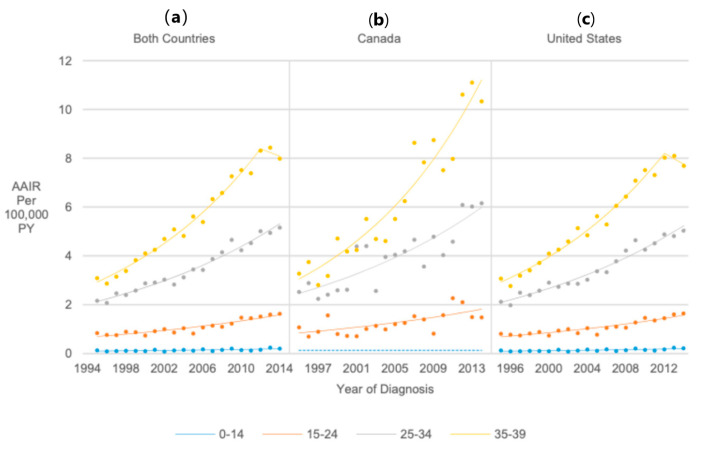
Joinpoint regression trends in age-adjusted incidence of thyroid cancer among male pediatrics, adolescents, and young adults in Canada/United States combined (**a**), Canada (**b**), and the United States (**c**) from 1995 to 2014 stratified by age at diagnosis.

**Figure 7:**
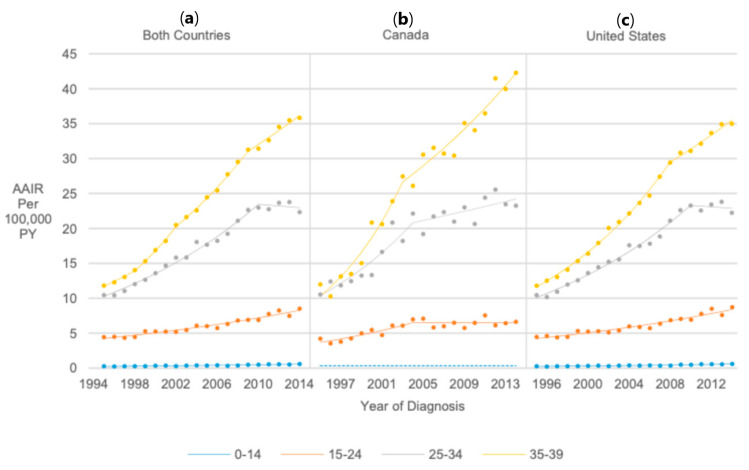
Joinpoint regression trends in age-adjusted incidence of thyroid cancer among female pediatrics, adolescents, and young adults in Canada/United States combined (**a**), Canada (**b**), and the United States (**c**) from 1995 to 2014 stratified by age at diagnosis.

When stratifying AAIR trends by sex and age in both countries combined, males (AAPC range 3.52–5.50) and females (AAPC range 3.59–6.12) of all age groups experienced similar relative AAIR increases from 1995 to 2014 (Figure 6 and Figure 7).

### 3.9. Race/Ethnicity/Sex (United States Only)

**Figure 8:**
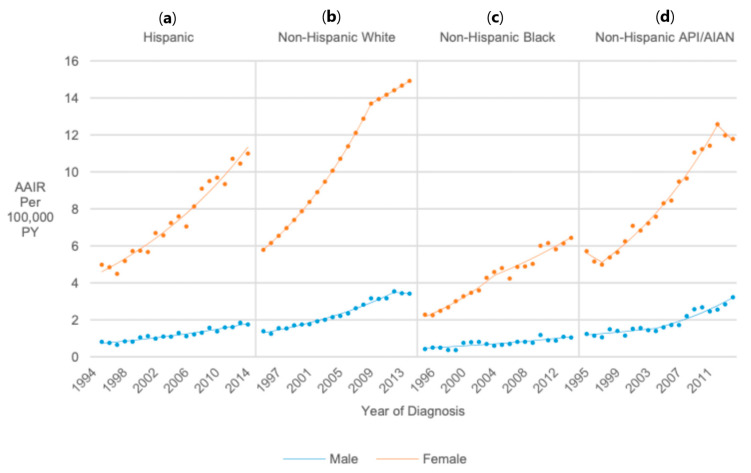
Joinpoint regression trends in age-adjusted incidence of thyroid cancer among pediatrics, adolescents, and young adults in the United States stratified by race/ethnicity and sex.

Males and females of all race/ethnicity groups experienced significant AAIR increases from 1995 to 2014 (AAPC range = 3.94–5.92) (Figure 8).

## 4. Discussion

This study is the first to describe incidence trends in thyroid cancer among the PAYA population of North America. We found a 137% increase in thyroid cancer incidence among this young population in the last two decades. Thyroid cancer incidence rates also increased when stratified by age at diagnosis, sex, and, in the United States, by race/ethnicity. Papillary thyroid cancer, the most common histology, was the predominant contributor to the increased incidence. Thyroid cancer incidence was higher in Canada than the United States for both males and females. Our results indicate an increasing incidence even among younger age groups, mirroring findings from other high-income countries in Europe and Asia [11]. These variations across different age groups and healthcare systems underscore the complexity of global thyroid cancer trends.

We observed a slower rise in thyroid cancer incidence in recent years in both the United States and Canada, similar to previous studies in older adult populations [6,8,10]. While we did not notice a significant difference in AAPCs between the United States and Canada overall, individuals from the ages of 25 to 34 and 35 to 39 tended to have higher incidence in Canada than the United States. The Canadian healthcare system provides universal healthcare access at no cost to the patient, which removes barriers to access and therefore might increase the risk of overdiagnosis. In the United States, non-Hispanic White individuals exhibited the highest incidence rates, while lower rates were seen among ethnically minoritized groups, likely attributed to poorer access to insurance and health care [26]. While limited healthcare access certainly has been associated with worse health outcomes, it is worth considering whether, in this context, it may also reduce the risk of overdiagnosis. The less drastic increase in incidence we saw around 2009 in the United States coincides with the ATA’s recommendation against fine needle aspiration for every thyroid nodule [27]. However, since 35 years old marks the threshold for recommended thyroid dysfunction screening per ATA guidelines [22,28], the large increase we saw in the 35–39 age group in our study may reflect potential overdiagnosis due to both thyroid-related screening and incidental findings on imaging ordered for other indications. In 2017, the United States Preventive Services Task Force (USPSTF) recommended against any routine thyroid cancer screening [29], and a recent study suggested that physicians who did routine TSH and cancer tests were more likely to diagnose thyroid cancer [30]. We are beginning to see plateaus in thyroid cancer incidence in the United States, potentially as an effect of policy changes influencing diagnostic pressure, though incidence remaining at high levels still suggests overdiagnosis is a public health issue requiring attention [16].

Pediatric and younger adults are least likely to be exposed to asymptomatic screening, as most cases of thyroid cancer are diagnosed in individuals 40 years of age or older [9]. However, we found an increased incidence in all age groups in both the United States and Canada. This is most significant in the 0–14 age group, who should have the least risk of being inappropriately screened. The increasing incidence of thyroid cancer among PAYA populations raises concerns about overdiagnosis as a significant contributing factor. Overdiagnosis refers to the detection of cancers that would not have caused symptoms or harm during a patient’s lifetime [4,11]. In the context of thyroid cancer, this phenomenon has been linked to the widespread use of advanced diagnostic tools, such as high-resolution ultrasonography and fine-needle aspiration biopsies, leading to the identification of subclinical, indolent tumors [11,31]. Additional factors like access to healthcare, and therefore imaging, as well as physician practice preferences also contribute to overdiagnosis [7]. As higher resolution imaging by ultrasound, CT, and MRI has become more widely used, thyroid masses may be incidental findings in imaging of the head and neck or chest, and lead to further investigation. These “incidentalomas” may be a large proportion of the “cancers” found. In both the United States and Canada, overdiagnosis of subclinical thyroid tumors has been identified as a problem that puts PAYA populations in particular at risk of overtreatment [4,8].

While much of the recent literature has suggested that the increasing incidence of thyroid cancer is partly due to overdiagnosis [3,7,10,32], other studies explored additional causes. In Canada, Harper et al. (2023) identify thyroid cancer as the primary driver of increasing AYA cancer incidence, highlighting its growing public health relevance [33]. However, recent data suggest that this trend may be shifting, particularly among female patients. Norwood et al. (2020) reports a decline in thyroid cancer rates among females in Ontario, mirroring patterns observed in the United States [34]. This decline may be attributed to the 2015 guideline changes discouraging the evaluation of sub-centimeter thyroid nodules, thereby reducing overdiagnosis [34,35]. Despite this, age–period–cohort effects continue to influence non-incidentally detected thyroid cancer cases, aligning with previous findings in Canada [34]. Liu et al. (2001) further supports the role of cohort and period effects in shaping thyroid cancer incidence, demonstrating that increases observed between 1970 and 1996 were largely attributable to birth cohort effects in males and both birth cohort and period effects in females [36]. These patterns suggest that environmental or behavioral changes over time, such as increased childhood and adolescent radiation exposure, may have contributed to rising thyroid cancer rates, particularly in Canada [36]. Collectively, these findings indicate that while overdiagnosis has played a role, other etiological factors, including radiation exposure and generational risk shifts, may be influencing thyroid cancer trends in AYA populations. Recent population-based studies from the United States also saw increased incidence rates of all sizes of pediatric thyroid cancer, of which the larger tumors were unlikely to have been found incidentally [9,19,37]. Including our findings across the PAYA age spectrum of North America, these findings all question whether medical surveillance or overdiagnosis alone is the driver of the persistently increasing incidence seen in thyroid cancer. Studies of the pathway to diagnosis for these cancers may elucidate the reasons better, and therefore how to find those that are worth identifying while minimizing overdiagnosis.

Another proposed environmental factor associated with increased thyroid cancer incidence is obesity. Several studies, including meta-analyses, have described an association between increased BMI and thyroid cancer in both males and females, including young adult patients [38,39,40,41]. As the United States is currently experiencing an epidemic of childhood obesity [42], it is plausible that in addition to overdiagnosis, the increasing overweight and obesity rates may be an important contributing factor to the increased incidence of thyroid cancer seen in the PAYA population from the United States and other developed nations, including Canada [42,43]. Future studies should examine other factors that may be associated with the increasing incidence of thyroid cancer [44], including the potential role of estrogen [45].

### 4.1. Global Trends in Thyroid Cancer Incidence

Global comparisons in thyroid cancer incidence rates must be interpreted with nuance, as diagnostic and screening practices vary considerably by region. Similar to our observations, incidence has increased substantially in Europe, particularly in France and Italy, and even more dramatically in South Korea [11]. Countries such as the Republic of Korea have shown extremely high detections of subclinical thyroid nodules due to widespread ultrasonography [4,17], whereas more moderate increases in Japan and the Nordic countries suggest the less prominent role of imaging-driven overdiagnosis in those settings [3,11]. The North Africa and Middle East region reported an age-standardized incidence rate of 3.5 per 100,000 population [46]; the lower incidence rates in this region compared to other countries globally may result from underreporting, limited access to healthcare, and less widespread use of diagnostic tools. While studies examining thyroid cancer rates in migrant populations are limited, a study of Asian immigrant populations in the United States suggests that incidence patterns are associated with birthplace, highlighting the importance of environmental and healthcare access factors in driving thyroid cancer risk [47]. Considering pediatric populations, a recent global meta-analysis of pediatric thyroid cancer found that while differentiated thyroid carcinoma remains rare in children and adolescents, incidence has been rising worldwide, particularly among Caucasian females [48]. Our study addresses a major limitation of prior studies included in this meta-analysis by providing detailed rates by sex and age group [48]. Their findings align with our observations in PAYA populations, reinforcing the need to understand how overdiagnosis and other social and environmental influences contribute to increasing incidence.

### 4.2. Clinical and Public Health Implications

The current study estimates that between 1995 and 2014, thyroid cancer incidence in North America increased annually by 4% among 0–14-year-olds, 4% among 15–24, 6% among 25–34-year-olds, and 4% among 35–39-year-olds. While increases were mostly associated with papillary thyroid cancer, there were also minor increases in medullary and follicular thyroid carcinomas. The disproportionate increase in papillary thyroid cancer incidence compared to other thyroid cancer subtypes likely reflects a combination of increased subclinical detection, its characteristically indolent course, histological reclassification, and distinct environmental influences like childhood radiation exposure [3,8,10]. Papillary thyroid cancer is the most easily identified thyroid malignancy through fine-needle aspiration and ultrasonography, contributing to its increasing detection rate, especially for small subclinical tumors [3]. Additionally, histological reclassification has led to increased diagnoses of follicular-variant papillary thyroid carcinoma, further driving incidence trends [10]. Overdiagnosis and overtreatment in the face of diagnostic uncertainty can have major life-long implications for comorbidity in overtreated PAYA patients, including unnecessary surgery, hormone replacement therapy, and the costs associated with surveillance [49].

### 4.3. Strengths and Limitations

This study has some important limitations. First, while we described trends in the incidence of thyroid cancer over two decades, the descriptive, retrospective nature of our data means we cannot ascribe causality to the factors described. Second, our analysis is limited by the use of registry data, which may be subject to regional differences in case ascertainment and completeness. However, we only used high-quality NAACCR Gold or Silver certified registries to ensure at least 90% case ascertainment completeness [50]. Third, our data do not provide information on thyroid cancer risk factors such as prior radiation exposure. While we were able to compare overall incidence in the United States versus Canada, we could not compare racial differences due to a lack of race information from the Canadian data.

Notwithstanding these limitations, our study makes an important contribution to the thyroid cancer epidemiology and surveillance literature in North America. It describes 20-year incidence trends in thyroid cancer, using the largest population-based data available, and shows the rising incidence of thyroid cancer in the PAYA population of North America. In light of the USPSTF recommendation against thyroid cancer screening [29], future studies should monitor how the incidence of thyroid cancer changes with changes in medical procedures and recommendations. Finally, future research should continue to analyze incidence trends beyond our study period, comparing patterns between the United States and Canada, and should incorporate individual-level pathology data such as tumor stage, histologic subtype, and extent of disease at initial surgery to better understand the factors driving these trends.

## 5. Conclusions

We describe 20-year incidence trends and show the rising incidence of thyroid cancer in the PAYA population of North America, similar to those found among older segments of the population. This upward trend was consistent across all age groups, with the most pronounced increases observed in older young adults. Incidence was higher among females and individuals in Canada compared to the United States, and among non-Hispanic White individuals compared to other racial and ethnic groups in the United States. This suggests that access to care is a strong driving factor, leading to the hypothesis that overdiagnosis is a large component of the increase in incidence rates. While evidence suggests that overdiagnosis—driven by increased incidental imaging, physicians’ overcalling of thyroid tumors, and disparities in healthcare access—primarily accounts for this trend, other contributing factors cannot be ruled out. Given the risk for unnecessary thyroidectomy and lifelong hormone replacement, it is crucial to reassess the role of incidental imaging to prevent avoidable medical interventions and long-term patient harm in the PAYA population. Finally, continuing research and surveillance of the drivers of rising thyroid cancer incidence is critical.

## Figures and Tables

**Table 1:** Thyroid cancer patients ages 0–39 diagnosed between 1995 and 2014, NAACCR CiNA Public Use Database.

	Canada and United States Combined			Canada			United States		
	Count (%)	Age-Adjusted Incidence Rate per 100,000 Person-Years (95% Confidence Interval)	Rate Ratio (95% Confidence Interval)	Count (%)	Age-Adjusted Incidence Rate per 100,000 Person-Years (95% Confidence Interval)	Rate Ratio (95% Confidence Interval)	Count (%)	Age-Adjusted Incidence Rate per 100,000 Person-Years (95% Confidence Interval)	Rate Ratio (95% Confidence Interval)
**Overall**	133,808	5.48 (5.45, 5.51)	-	15,492	5.94 (5.85, 6.04)	-	118,316	5.42 (5.39, 5.45)	-
**Age**									
0–14	2306 (1.7%)	0.26 (0.25, 0.27)	Reference	201 (1.3%)	0.24 (0.20, 0.27)	Reference	2105 (1.8%)	0.26 (0.25, 0.27)	Reference
15–24	22,036 (16.5%)	3.55 (3.50, 3.59)	13.73 (13.15, 14.34)	2213 (14.3%)	3.47 (3.33, 3.62)	14.71 (12.72, 17.08)	19,823 (16.8%)	3.56 (3.51, 3.60)	13.63 (13.03, 14.27)
25–34	63,427 (47.4%)	10.42 (10.34, 10.50)	40.33 (38.69, 42.06)	7377 (47.6%)	11.37 (11.11, 11.64)	48.23 (41.91, 55.76)	56,050 (47.4%)	10.30 (10.22, 10.39)	39.51 (37.83, 41.29)
35–39	46,039 (34.4%)	14.38 (14.25, 14.51)	55.66 (53.38, 58.06)	5701 (36.8%)	16.25 (15.83, 16.68)	68.91 (59.85, 79.72)	40,338 (34.1%)	14.15 (14.01, 14.29)	54.26 (51.93, 56.71)
**Sex**									
Male	23,520 (17.6%)	1.92 (1.89, 1.94)	Reference	2830 (18.3%)	2.17 (2.09, 2.25)	Reference	20,690 (17.5%)	1.89 (1.86, 1.92)	Reference
Female	110,288 (82.4%)	9.10 (9.04, 9.15)	4.74 (4.67, 4.81)	12,662 (81.7%)	9.76 (9.59, 9.93)	4.51 (4.32, 4.69)	97,626 (82.5%)	9.02 (8.96, 9.07)	4.77 (4.70, 4.84)
**Histology**									
Papillary	120,433 (90.0%)	4.93 (4.90, 4.96)	Reference	13,966 (90.1%)	5.36 (5.27, 5.45)	Reference	106,467 (90.0%)	4.88 (4.85, 4.91)	Reference
Follicular	9232 (6.9%)	0.38 (0.37, 0.38)	0.08 (0.07, 0.08)	665 (4.3%)	0.26 (0.24, 0.28)	0.05 (0.04, 0.05)	8567 (7.2%)	0.39 (0.38, 0.40)	0.08 (0.08, 0.08)
Medullary	1802 (1.3%)	0.07 (0.07, 0.08)	0.02 (0.01, 0.02)	183 (1.2%)	0.07 (0.06, 0.08)	0.01 (0.01, 0.02)	1619 (1.4%)	0.07 (0.07, 0.08)	0.02 (0.01, 0.02)
Anaplastic/other	1450 (1.1%)	0.06 (0.06, 0.06)	0.01 (0.01, 0.01)	103 (0.7%)	0.04 (0.03, 0.05)	0.01 (0.01, 0.01)	1347 (1.1%)	0.06 (0.06, 0.07)	0.01 (0.01, 0.01)
Non microscopically confirmed	891 (0.7%)	0.04 (0.03, 0.04)	0.01 (0.01, 0.01)	575 (3.7%)	0.22 (0.20, 0.24)	0.04 (0.04, 0.04)	316 (0.3%)	0.01 (0.01, 0.02)	0.003 (0.003, 0.003)
**Race/ethnicity**									
Non-Hispanic White				-			80,507 (68.0%)	6.22 (6.18, 6.27)	Reference
Hispanic				-			20,767 (17.6%)	4.38 (4.32, 4.44)	0.70 (0.69, 0.71)
Non-Hispanic Black				-			6483 (5.5%)	2.59 (2.52, 2.65)	0.42 (0.41, 0.43)
Non-Hispanic Other				-			8733 (7.4%)	5.43 (5.32, 5.55)	0.87 (0.85, 0.89)
Unknown				-			1826 (1.5%)	-	-

**Table 2:** Annual percentage changes (APC) and average APCs (AAPC) based on Joinpoint regression trends in age-adjusted incidence of thyroid cancer among pediatrics, adolescents, and young adults in Canada, the United States, and both countries combined from 1995 to 2014.

	Both Countries			Canada			United States		
	Year Range	APC (95% CI)	1995–2014 AAPC (95% CI)	Year Range	APC (95% CI)	1995–2014 AAPC (95% CI)	Year Range	APC (95% CI)	1995–2014 AAPC (95% CI)
**Overall**	1995–2009	5.87 (5.51, 6.24)	4.95 (4.54, 5.36)	1995–1998	2.69 (−5.40, 11.47)	5.11 (3.21, 7.05)	1995–2010	5.73 (5.41, 6.07)	4.87 (4.43, 5.32)
	2009–2014	2.42 (1.11, 3.75)	-	1998–2002	12.60 (4.82, 20.96)		2010–2014	1.71 (−0.15, 3.60)	
				2002–2014	3.32 (2.63, 4.02)				
**Age**									
0–14	1995–2014	4.28 (3.36, 5.20)	4.28 (3.36, 5.20)	1995–2014	Not calculated-insufficient data	-	1995–2014	4.56 (3.58, 5.54)	4.56 (3.58, 5.54)
15–24	1995–2014	3.72 (3.34, 4.10)	3.72 (3.34, 4.10)	1995–2014	2.90 (1.87, 3.95)	2.90 (1.87, 3.95)	1995–2014	3.81 (3.38, 4.24)	3.81 (3.38, 4.24)
25–34	1995–2009	5.77 (5.30, 6.24)	4.53 (4.01, 5.06)	1995–2002	8.89 (4.92, 13.01)	4.93 (3.40, 6.47)	1995–2010	5.67 (5.22, 6.13)	4.47 (3.86, 5.08)
	2009–2014	1.16 (−0.50, 2.85)	-	2002–2014	2.68 (1.37, 4.01)		2010–2014	0.08 (−2.43, 2.66)	
35–39	1995–1997	4.17 (−4.72, 13.89)	5.99 (4.84, 7.15)	1995–2003	11.35 (8.65, 14.12)	7.61 (6.42, 8.81)	1995–2009	7.13 (6.77, 7.48)	5.91 (5.51, 6.30)
	1997–2002	9.06 (6.42, 11.77)	-	2003–2014	4.97 (3.83, 6.12)		2009–2014	2.56 (1.31, 3.83)	
	2002–2009	6.41 (5.25, 7.57)	-						
	2009–2014	3.15 (1.85, 4.47)	-						
**Sex**									
Male	1995–2014	5.22 (4.88, 5.55)	5.22 (4.88, 5.55)	1995–2014	5.50 (4.65, 6.35)	5.50 (4.65, 6.35)	1995–2014	5.17 (4.81, 5.53)	5.17 (4.81, 5.53)
Female	1995–2009	5.92 (5.53, 6.31)	4.89 (4.45, 5.32)	1995–1999	5.46 (0.54, 10.61)	5.17 (3.03, 7.37)	1995–2009	5.89 (5.54, 6.25)	4.91 (4.51, 5.31)
	2009–2014	2.04 (0.65, 3.45)	-	1999–2002	15.38 (1.50, 31.16)		2009–2014	2.21 (0.94, 3.49)	
				2002–2014	2.68 (2.04, 3.31)				
**Histology**									
Papillary	1995–2009	6.49 (6.09, 6.89)	5.50 (5.06, 5.94)	1995–2004	9.13 (7.27, 11.02)	6.44 (5.48, 7.41)	1995–2009	6.41 (6.02, 6.80)	5.44 (5.02, 5.87)
	2009–2014	2.78 (1.40, 4.19)		2004–2014	4.08 (2.97, 5.20)		2009–2014	2.78 (1.43, 4.15)	
Follicular	1995–2006	2.00 (1.52, 2.49)	0.21 (−0.62, 1.05)	1995–2014	−0.45 (−1.66, 0.77)	−0.45 (−1.66, 0.77)	1995–2006	2.06 (1.51, 2.62)	0.23 (−0.72, 1.18)
	2006–2012	−0.55 (−2.03, 0.96)					2006–2012	−0.31 (−2.00, 1.41)	
	2012–2014	−7.00 (−13.33, −0.21)					2012–2014	−7.81 (−14.95, −0.06)	
Medullary	1995–2014	1.16 (0.47, 1.85)	1.16 (0.47, 1.85)	1995–2014	Not calculated-insufficient data	-	1995–2014	1.21 (0.49, 1.94)	1.21 (0.49, 1.94)
Anaplastic/Other	1995–2014	1.94 (0.68, 3.22)	1.94 (0.68, 3.22)	1995–2014	Not calculated-insufficient data	-	1995–2014	2.15 (0.89, 3.42)	2.15 (0.89, 3.42)
Not microscopically confirmed	1995–2003	6.62 (0.45, 13.18)	−5.60 (−8.97, −2.11)	1995–2014	Not calculated-insufficient data	-	1995–2014	−1.07 (−3.08, 0.97)	−1.07 (−3.08, 0.97)
	2003–2014	−13.60 (−18.03, −8.92)							
**Race/Ethnicity**									
Hispanic	-			-			1995–2014	4.99 (4.56, 5.43)	4.99 (4.56, 5.43)
Non-Hispanic White	-			-			1995–2009	6.30 (5.96, 6.64)	5.16 (4.77, 5.55)
							2009–2014	2.03 (0.77, 3.30)	
Non-Hispanic Black	-			-			1995–2003	8.36 (5.89, 10.88)	5.81 (4.70, 6.94)
							2003–2014	4.00 (2.88, 5.13)	
Non-Hispanic API/AIAN	-			-			1995–1997	−3.29 (−17.08, 12.79)	4.37 (2.22, 6.56)
							1997–2006	5.47 (4.00, 6.96)	
							2006–2009	9.83 (−0.51, 21.25)	
							2009–2014	2.40 (0.46, 4.37)	
**Sex/Age**									
Male 0–14	1995–2014	3.52 (1.72, 5.34)	3.52 (1.72, 5.34)	1995–2014	Not calculated-insufficient data	-	1995–2014	3.94 (2.10, 5.81)	3.94 (2.10, 5.81)
Male 15–24	1995–2014	4.40 (3.58, 5.22)	4.40 (3.58, 5.22)	1995–2014	4.10 (1.85, 6.40)	4.10 (1.85, 6.40)	1995–2014	4.43 (3.56, 5.30)	4.43 (3.56, 5.30)
Male 25–34	1995–2014	4.94 (4.50, 5.38)	4.94 (4.50, 5.38)	1995–2014	4.78 (3.42, 6.17)	4.78 (3.42, 6.17)	1995–2014	4.94 (4.43, 5.45)	4.94 (4.43, 5.45)
Male 35–39	1995–2012	6.40 (5.85, 6.95)	5.50 (4.25, 6.77)	1995–2014	7.09 (5.95, 8.24)	7.09 (5.95, 8.24)	1995–2012	6.27 (5.71, 6.84)	5.28 (3.97, 6.61)
	2012–2014	−1.84 (−12.54, 10.18)					2012–2014	−2.79 (−13.89, 9.74)	
Female 0–14	1995–2014	4.60 (3.76, 5.45)	4.60 (3.76, 5.45)	1995–2014	Not calculated-insufficient data		1995–2014	4.80 (3.95, 5.66)	4.80 (3.95, 5.66)
Female 15–24	1995–2014	3.59 (3.17, 4.00)	3.59 (3.17, 4.00)	1995–2004	6.54 (3.48, 9.69)	3.03 (1.40, 4.69)	1995–2014	3.69 (3.23, 4.15)	3.69 (3.23, 4.15)
				2004–2014	−0.03 (−2.01, 1.98)				
Female 25–34	1995–2010	5.66 (5.19, 6.14)	4.32 (3.69, 4.96)	1995–2004	8.03 (5.39, 10.73)	4.55 (3.15, 5.97)	1995–2010	5.73 (5.25, 6.21)	4.40 (3.75, 5.05)
	2010–2014	−0.54 (−3.16, 2.14)		2004–2014	1.52 (−0.18, 3.24)		2010–2014	−0.46 (−3.12, 2.27)	
Female 35–39	1995–1998	5.98 (2.87, 9.18)	6.12 (5.39, 6.86)	1995–2003	12.61 (9.59, 15.71)	7.70 (6.38, 9.03)	1995–2008	7.44 (7.08, 7.80)	6.07 (5.73, 6.40)
	1998–2002	9.81 (7.02, 12.67)		2003–2014	4.26 (3.04, 5.51)		2008–2014	3.16 (2.32, 4.00)	
	2002–2009	6.30 (5.53, 7.08)							
	2009–2014	3.09 (2.21, 3.98)							
**Race/Sex**									
Hispanic Male	-			-			1995–2014	4.81 (4.09, 5.54)	4.81 (4.09, 5.54)
Hispanic Female	-			-			1995–2014	4.86 (4.40, 5.34)	4.86 (4.40, 5.34)
Non-Hispanic White Male	-			-			1995–2012	5.99 (5.46, 6.52)	5.22 (3.96, 6.49)
							2012–2014	−1.13 (−12.05, 11.14)	
Non-Hispanic White Female	-			-			1995–2009	6.34 (5.99, 6.69)	5.11 (4.71, 5.51)
							2009–2014	1.73 (0.43, 3.04)	
Non-Hispanic Black Male	-			-			1995–2014	4.49 (2.96, 6.05)	4.49 (2.96, 6.05)
Non-Hispanic Black Female	-			-			1995–2004	8.19 (6.29, 10.13)	5.92 (4.91, 6.94)
							2004–2014	3.92 (2.72, 5.13)	
Non-Hispanic API/AIAN Male	-			-			1995–2004	2.94 (−0.56, 6.57)	5.36 (3.48, 7.28)
							2004–2014	7.59 (5.43, 9.80)	
Non-Hispanic API/AIAN Female	-			-			1995–1997	−4.53 (−18.25, 11.49)	3.94 (2.18, 5.74)
							1997–2012	6.15 (5.58, 6.72)	
							2012–2014	−3.31 (−11.12, 5.19)	

## Data Availability

The original data presented in the study are openly available in North American Association of Central Cancer Registries’ (NAACCR) Cancer in North America (CiNA) Public Use dataset at [https://www.naaccr.org/cina-public-use-data-set/] (accessed on 1 February 2025).

## Supplementary Materials

The following supporting information can be downloaded at: https://www.mdpi.com/article/10.3390/cancers17091429/s1, Table S1: Differences in average annual percentage changes (AAPCs) and parallelism tests between variable value pairs within Canada, the United States, and both countries combined; Table S2: Differences in average annual percentage changes (AAPCs) and parallelism tests between Canada and the United States overall and for specific variable values.

## References

[1] American Cancer Society (2024). Cancer Facts & Figures 2024.

[2] Canadian Cancer Statistics Advisory Committee (2018). Canadian Cancer Statistics 2018.

[3] Vaccarella S., Franceschi S., Bray F., Wild C.P., Plummer M., Dal Maso L. (2016). Worldwide Thyroid-Cancer Epidemic? The Increasing Impact of Overdiagnosis. N. Engl. J. Med..

[4] Kitahara C.M., Sosa J.A. (2016). The changing incidence of thyroid cancer. Nat. Rev. Endocrinol..

[5] Zanocco K.A., Hershman J.M., Leung A.M. (2019). Active Surveillance of Low-Risk Thyroid Cancer. JAMA.

[6] Davies L., Welch H.G. (2014). Current thyroid cancer trends in the United States. JAMA Otolaryngol.—Head Neck Surg..

[7] Sanabria A., Kowalski L.P., Shah J.P., Nixon I.J., Angelos P., Williams M.D., Rinaldo A., Ferlito A. (2018). Growing incidence of thyroid carcinoma in recent years: Factors underlying overdiagnosis. Head Neck.

[8] Topstad D., Dickinson J.A. (2017). Thyroid cancer incidence in Canada: A national cancer registry analysis. CMAJ Open.

[9] Lim H., Devesa S.S., Sosa J.A., Check D., Kitahara C.M. (2017). Trends in Thyroid Cancer Incidence and Mortality in the United States, 1974–2013. JAMA.

[10] Enewold L., Zhu K., Ron E., Marrogi A.J., Stojadinovic A., Peoples G.E., Devesa S.S. (2009). Rising thyroid cancer incidence in the United States by demographic and tumor characteristics, 1980–2005. Cancer Epidemiol. Biomark. Prev..

[11] Vaccarella S., Dal Maso L., Laversanne M., Bray F., Plummer M., Franceschi S. (2015). The Impact of Diagnostic Changes on the Rise in Thyroid Cancer Incidence: A Population-Based Study in Selected High-Resource Countries. Thyroid.

[12] Coccia P.F. (2019). Overview of Adolescent and Young Adult Oncology. J. Oncol. Pract..

[13] Ladenson P.W., Singer P.A., Ain K.B., Bagchi N., Bigos S.T., Levy E.G., Smith S.A., Daniels G.H., Cohen H.D. (2000). American Thyroid Association guidelines for detection of thyroid dysfunction. Arch. Intern. Med..

[14] Canadian Task Force on the Periodic Health Examination, Canada. Health Canada (1994). The Canadian Guide to Clinical Preventive Health Care.

[15] Dal Maso L., Lise M., Zambon P., Falcini F., Crocetti E., Serraino D., Cirilli C., Zanetti R., Vercelli M., Ferretti S. (2011). Incidence of thyroid cancer in Italy, 1991-2005: Time trends and age-period-cohort effects. Ann. Oncol..

[16] Chen M.M., Luu M., Sacks W.L., Orloff L., Wallner L.P., Clair J.M., Pitt S.C., Ho A.S., Zumsteg Z.S. (2025). Trends in incidence, metastasis, and mortality from thyroid cancer in the USA from 1975 to 2019: A population-based study of age, period, and cohort effects. Lancet Diabetes Endocrinol..

[17] Ahn H.S., Kim H.J., Welch H.G. (2014). Korea’s thyroid-cancer “epidemic”—Screening and overdiagnosis. N. Engl. J. Med..

[18] Siegel D.A., King J., Tai E., Buchanan N., Ajani U.A., Li J. (2014). Cancer incidence rates and trends among children and adolescents in the United States, 2001–2009. Pediatrics.

[19] Qian Z.J., Jin M.C., Meister K.D., Megwalu U.C. (2019). Pediatric Thyroid Cancer Incidence and Mortality Trends in the United States, 1973–2013. JAMA Otolaryngol.—Head Neck Surg..

[20] North American Association of Central Cancer Registries Description of CiNA Public Use Dataset. https://www.naaccr.org/wp-content/uploads/2017/09/Description-of-CiNA-Public-Use-Dataset.pdf.

[21] Tomar S.L., Loree M., Logan H. (2004). Racial differences in oral and pharyngeal cancer treatment and survival in Florida. Cancer Causes Control.

[22] Garber J.R., Cobin R.H., Gharib H., Hennessey J.V., Klein I., Mechanick J.I., Pessah-Pollack R., Singer P.A., Woeber K.A. (2012). Clinical Practice Guidelines for Hypothyroidism in Adults: Cosponsored by the American Association of Clinical Endocrinologists and the American Thyroid Association. Thyroid.

[23] Anderson R.N., Rosenberg H.M. (1998). Age standardization of death rates: Implementation of the year 2000 standard. Natl. Vital Stat. Rep..

[24] National Cancer Institute Linear or Log-Linear Model. https://surveillance.cancer.gov/help/joinpoint/tech-help/frequently-asked-questions/linear-or-log-linear-model.

[25] National Cancer Institute Selecting the Final Model. https://surveillance.cancer.gov/help/joinpoint/tech-help/frequently-asked-questions/selecting-the-final-model.

[26] Gillis A., Chen H., Wang T.S., Dream S. (2024). Racial and Ethnic Disparities in the Diagnosis and Treatment of Thyroid Disease. J. Clin. Endocrinol. Metab..

[27] Cooper D.S., Doherty G.M., Haugen B.R., Kloos R.T., Lee S.L., Mandel S.J., Mazzaferri E.L., McIver B., Pacini F., Schlumberger M. (2009). Revised American Thyroid Association management guidelines for patients with thyroid nodules and differentiated thyroid cancer. Thyroid.

[28] Rugge B., Balshem H., Sehgal R., Relevo R., Gorman P., Helfand M. (2011). Screening and Treatment of Subclinical Hypothyroidism or Hyperthyroidism.

[29] Bibbins-Domingo K., Grossman D.C., Curry S.J., Barry M.J., Davidson K.W., Doubeni C.A., Epling J.W., Kemper A.R., Krist A.H., Kurth A.E. (2017). Screening for Thyroid Cancer: US Preventive Services Task Force Recommendation Statement. JAMA.

[30] Hall S.F., Webber C., Groome P.A., Booth C.M., Nguyen P., DeWit Y. (2019). Do doctors who order more routine medical tests diagnose more cancers? A population-based study from Ontario Canada. Cancer Med..

[31] Dal Maso L., Vaccarella S., Franceschi S. (2025). Trends in thyroid cancer incidence and overdiagnosis in the USA. Lancet Diabetes Endocrinol..

[32] Hoang J.K., Nguyen X.V., Davies L. (2015). Overdiagnosis of thyroid cancer: Answers to five key questions. Acad. Radiol..

[33] Harper A.S., Diaz R.L., Cortez S.N.R., Shack L., Amin K., Bu J.V., Barr R.D., Fidler-Benaoudia M.M. (2023). Trends in the Incidence of Cancer Among Adolescent and Young Adults in Alberta, 1983–2017: A Population-Based Study Using Cancer Registry Data. J. Adolesc Young Adult Oncol..

[34] Norwood T.A., Buajitti E., Lipscombe L.L., Stukel T.A., Rosella L.C. (2020). Incidental detection, imaging modalities and temporal trends of differentiated thyroid cancer in Ontario: A population-based retrospective cohort study. CMAJ Open.

[35] Haugen B.R., Alexander E.K., Bible K.C., Doherty G.M., Mandel S.J., Nikiforov Y.E., Pacini F., Randolph G.W., Sawka A.M., Schlumberger M. (2016). 2015 American Thyroid Association Management Guidelines for Adult Patients with Thyroid Nodules and Differentiated Thyroid Cancer: The American Thyroid Association Guidelines Task Force on Thyroid Nodules and Differentiated Thyroid Cancer. Thyroid.

[36] Liu S., Semenciw R., Ugnat A.M., Mao Y. (2001). Increasing thyroid cancer incidence in Canada, 1970–1996: Time trends and age-period-cohort effects. Br. J. Cancer.

[37] Bernier M.O., Withrow D.R., Berrington de Gonzalez A., Lam C.J.K., Linet M.S., Kitahara C.M., Shiels M.S. (2019). Trends in pediatric thyroid cancer incidence in the United States, 1998–2013. Cancer.

[38] Ma J., Huang M., Wang L., Ye W., Tong Y., Wang H. (2015). Obesity and risk of thyroid cancer: Evidence from a meta-analysis of 21 observational studies. Med. Sci. Monit..

[39] Colditz G.A., Peterson L.L. (2018). Obesity and Cancer: Evidence, Impact, and Future Directions. Clin. Chem..

[40] Kitahara C.M., Platz E.A., Freeman L.E., Hsing A.W., Linet M.S., Park Y., Schairer C., Schatzkin A., Shikany J.M., Berrington de Gonzalez A. (2011). Obesity and thyroid cancer risk among U.S. men and women: A pooled analysis of five prospective studies. Cancer Epidemiol. Biomark. Prev. A Publ. Am. Assoc. Cancer Res. Cosponsored Am. Soc. Prev. Oncol..

[41] Kitahara C.M., Pfeiffer R.M., Sosa J.A., Shiels M.S. (2019). Impact of overweight and obesity on U.S. papillary thyroid cancer incidence trends (1995–2015). J. Natl. Cancer Inst..

[42] Lauby-Secretan B., Scoccianti C., Loomis D., Grosse Y., Bianchini F., Straif K. (2016). Body Fatness and Cancer--Viewpoint of the IARC Working Group. N. Engl. J. Med..

[43] Sung H., Siegel R.L., Rosenberg P.S., Jemal A. (2019). Emerging cancer trends among young adults in the USA: Analysis of a population-based cancer registry. Lancet Public Health.

[44] Goldenberg D. (2019). We cannot ignore the real component of the rise in thyroid cancer incidence. Cancer.

[45] Zane M., Parello C., Pennelli G., Townsend D.M., Merigliano S., Boscaro M., Toniato A., Baggio G., Pelizzo M.R., Rubello D. (2017). Estrogen and thyroid cancer is a stem affair: A preliminary study. Biomed. Pharmacother..

[46] Nejadghaderi S.A., Moghaddam S.S., Azadnajafabad S., Rezaei N., Rezaei N., Tavangar S.M., Jamshidi H., Mokdad A.H., Naghavi M., Farzadfar F. (2022). Burden of thyroid cancer in North Africa and Middle East 1990–2019. Front. Oncol..

[47] Rossing M.A., Schwartz S.M., Weiss N.S. (1995). Thyroid cancer incidence in Asian migrants to the United States and their descendants. Cancer Causes Control.

[48] Moleti M., Aversa T., Crisafulli S., Trifirò G., Corica D., Pepe G., Cannavò L., Di Mauro M., Paola G., Fontana A. (2023). Global incidence and prevalence of differentiated thyroid cancer in childhood: Systematic review and meta-analysis. Front. Endocrinol..

[49] Dedhia P.H., Saucke M.C., Long K.L., Doherty G.M., Pitt S.C. (2022). Physician Perspectives of Overdiagnosis and Overtreatment of Low-Risk Papillary Thyroid Cancer in the US. JAMA Netw. Open.

[50] North American Association of Central Cancer Registries (NAACCR) Certification Criteria. https://www.naaccr.org/certification-criteria/.

